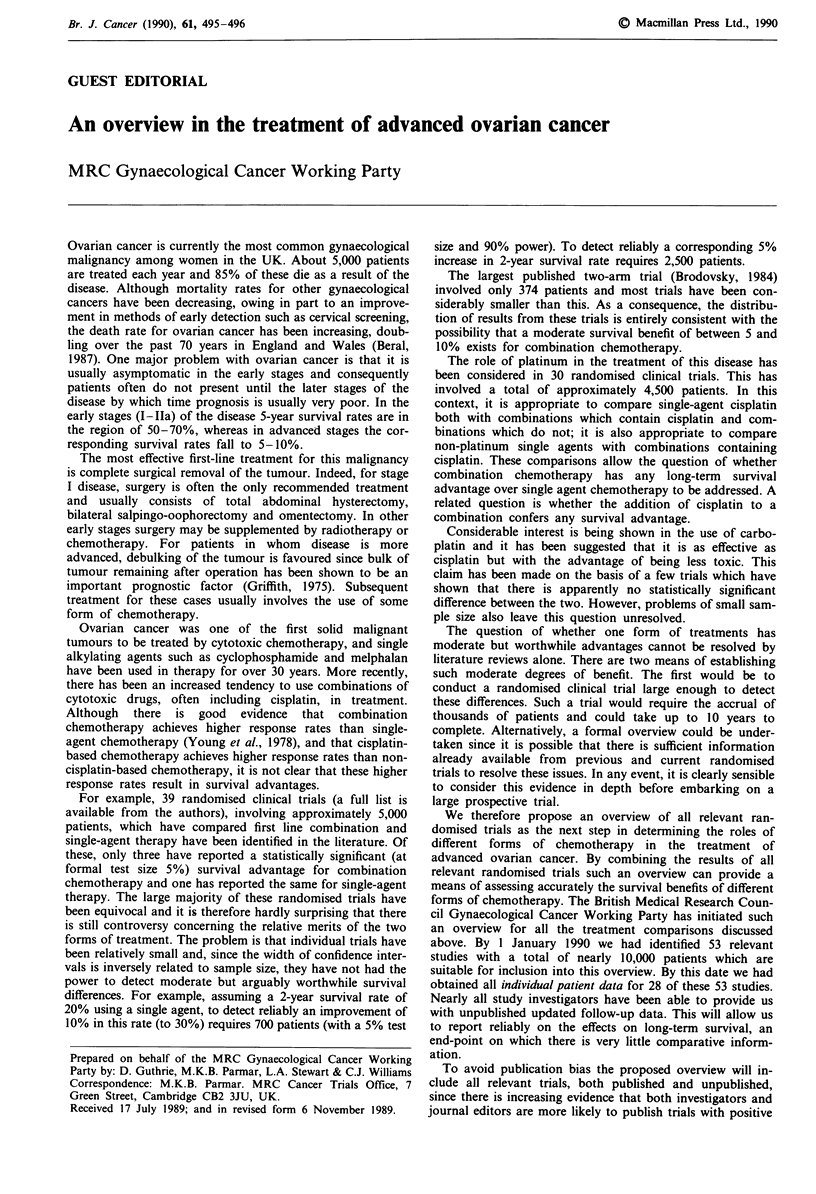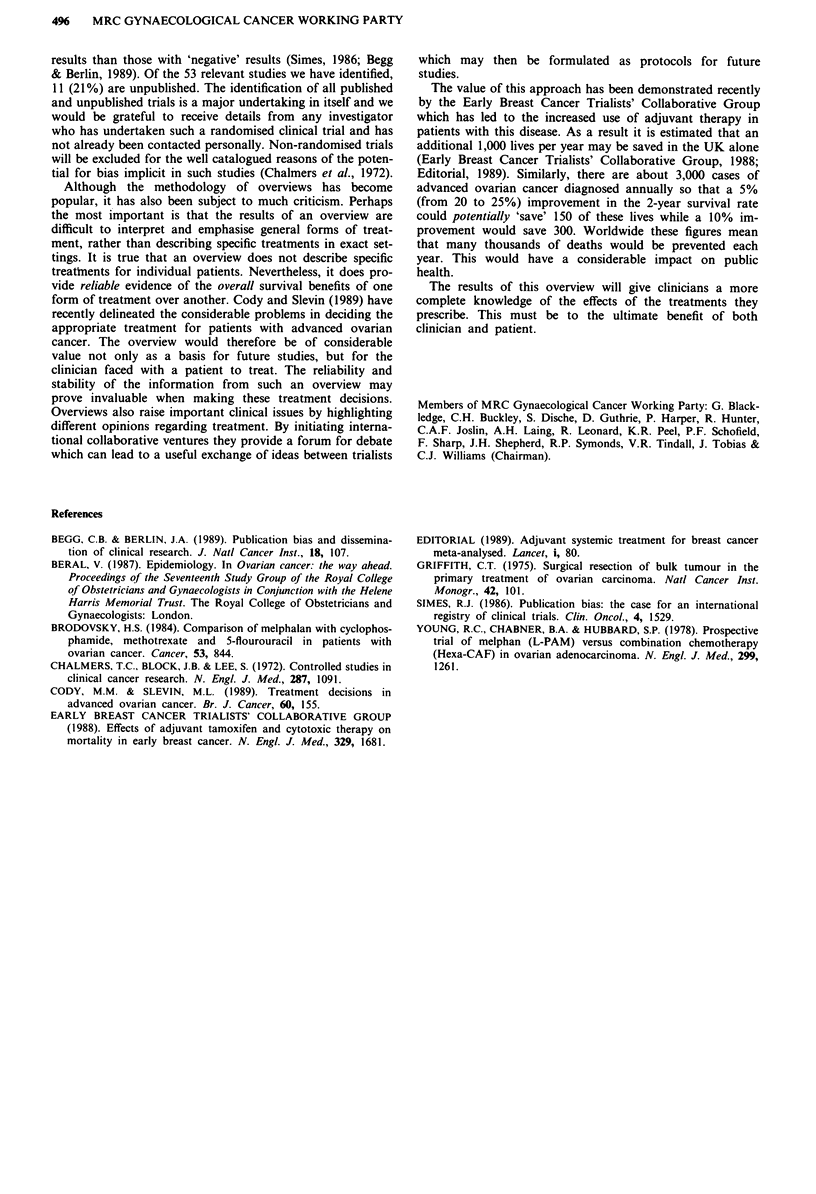# An overview in the treatment of advanced ovarian cancer. MRC Gynaecological Cancer Working Party.

**DOI:** 10.1038/bjc.1990.111

**Published:** 1990-04

**Authors:** 


Br. J. Cancer (1990), 61, 495-496 © Macmillan Press Ltd., 1990

GUEST EDITORIAL

### An overview in the treatment of advanced ovarian cancer

MRC Gynaecological Cancer Working Party

Ovarian cancer is currently the most common gynaecological malignancy among women in the UK. About 5,000 patients are treated each year and 85% of these die as a result of the disease. Although mortality rates for other gynaecological cancers have been decreasing, owing in part to an improvement in methods of early detection such as cervical screening, the death rate for ovarian cancer has been increasing, doubling over the past 70 years in England and Wales (Beral, 1987). One major problem with ovarian cancer is that it is usually asymptomatic in the early stages and consequently patients often do not present until the later stages of the disease by which time prognosis is usually very poor. In the early stages (I–IIa) of the disease 5-year survival rates are in the region of 50–70%, whereas in advanced stages the corresponding survival rates fall to 5–10%.

The most effective first-line treatment for this malignancy is complete surgical removal of the tumour. Indeed, for stage I disease, surgery is often the only recommended treatment and usually consists of total abdominal hysterectomy, bilateral salpingo-oophorectomy and omentectomy. In other early stages surgery may be supplemented by radiotherapy or chemotherapy. For patients in whom disease is more advanced, debulking of the tumour is favoured since bulk of tumour remaining after operation has been shown to be an important prognostic factor (Griffith, 1975). Subsequent treatment for these cases usually involves the use of some form of chemotherapy.

Ovarian cancer was one of the first solid malignant tumours to be treated by cytotoxic chemotherapy, and single alkylating agents such as cyclophosphamide and melphalan have been used in therapy for over 30 years. More recently, there has been an increased tendency to use combinations of cytotoxic drugs, often including cisplatin, in treatment. Although there is good evidence that combination chemotherapy achieves higher response rates than single-agent chemotherapy (Young *et al.*, 1978), and that cisplatin-based chemotherapy achieves higher response rates than non-cisplatin-based chemotherapy, it is not clear that these higher response rates result in survival advantages.

For example, 39 randomised clinical trials (a full list is available from the authors), involving approximately 5,000 patients, which have compared first line combination and single-agent therapy have been identified in the literature. Of these, only three have reported a statistically significant (at formal test size 5%) survival advantage for combination chemotherapy and one has reported the same for single-agent therapy. The large majority of these randomised trials have been equivocal and it is therefore hardly surprising that there is still controversy concerning the relative merits of the two forms of treatment. The problem is that individual trials have been relatively small and, since the width of confidence intervals is inversely related to sample size, they have not had the power to detect moderate but arguably worthwhile survival differences. For example, assuming a 2-year survival rate of 20% using a single agent, to detect reliably an improvement of 10% in this rate (to 30%) requires 700 patients (with a 5% test size and 90% power). To detect reliably a corresponding 5% increase in 2-year survival rate requires 2,500 patients.

The largest published two-arm trial (Brodovsky, 1984) involved only 374 patients and most trials have been considerably smaller than this. As a consequence, the distribution of results from these trials is entirely consistent with the possibility that a moderate survival benefit of between 5 and 10% exists for combination chemotherapy.

The role of platinum in the treatment of this disease has been considered in 30 randomised clinical trials. This has involved a total of approximately 4,500 patients. In this context, it is appropriate to compare single-agent cisplatin both with combinations which contain cisplatin and combinations which do not; it is also appropriate to compare non-platinum single agents with combinations containing cisplatin. These comparisons allow the question of whether combination chemotherapy has any long-term survival advantage over single agent chemotherapy to be addressed. A related question is whether the addition of cisplatin to a combination confers any survival advantage.

Considerable interest is being shown in the use of carboplatin and it has been suggested that it is as effective as cisplatin but with the advantage of being less toxic. This claim has been made on the basis of a few trials which have shown that there is apparently no statistically significant difference between the two. However, problems of small sample size also leave this question unresolved.

The question of whether one form of treatments has moderate but worthwhile advantages cannot be resolved by literature reviews alone. There are two means of establishing such moderate degrees of benefit. The first would be to conduct a randomised clinical trial large enough to detect these differences. Such a trial would require the accrual of thousands of patients and could take up to 10 years to complete. Alternatively, a formal overview could be undertaken since it is possible that there is sufficient information already available from previous and current randomised trials to resolve these issues. In any event, it is clearly sensible to consider this evidence in depth before embarking on a large prospective trial.

We therefore propose an overview of all relevant randomised trials as the next step in determining the roles of different forms of chemotherapy in the treatment of advanced ovarian cancer. By combining the results of all relevant randomised trials such an overview can provide a means of assessing accurately the survival benefits of different forms of chemotherapy. The British Medical Research Council Gynaecological Cancer Working Party has initiated such an overview for all the treatment comparisons discussed above. By 1 January 1990 we had identified 53 relevant studies with a total of nearly 10,000 patients which are suitable for inclusion into this overview. By this date we had obtained all *individual patient data* for 28 of these 53 studies. Nearly all study investigators have been able to provide us with unpublished updated follow-up data. This will allow us to report reliably on the effects on long-term survival, an end-point on which there is very little comparative information.

To avoid publication bias the proposed overview will include all relevant trials, both published and unpublished, since there is increasing evidence that both investigators and journal editors are more likely to publish trials with positive

Prepared on behalf of the MRC Gynaecological Cancer Working Party by: D. Guthrie, M.K.B. Parmar, L.A. Stewart & C.J. Williams
Correspondence: M.K.B. Parmar. MRC Cancer Trials Office, 7 Green Street, Cambridge CB2 3JU, UK.
Received 17 July 1989; and in revised form 6 November 1989.

results than those with 'negative' results (Simes, 1986; Begg & Berlin, 1989). Of the 53 relevant studies we have identified, 11 (21%) are unpublished. The identification of all published and unpublished trials is a major undertaking in itself and we would be grateful to receive details from any investigator who has undertaken such a randomised clinical trial and has not already been contacted personally. Non-randomised trials will be excluded for the well catalogued reasons of the potential for bias implicit in such studies (Chalmers *et al.*, 1972).

Although the methodology of overviews has become popular, it has also been subject to much criticism. Perhaps the most important is that the results of an overview are difficult to interpret and emphasise general forms of treatment, rather than describing specific treatments in exact settings. It is true that an overview does not describe specific treatments for individual patients. Nevertheless, it does provide *reliable* evidence of the *overall* survival benefits of one form of treatment over another. Cody and Slevin (1989) have recently delineated the considerable problems in deciding the appropriate treatment for patients with advanced ovarian cancer. The overview would therefore be of considerable value not only as a basis for future studies, but for the clinician faced with a patient to treat. The reliability and stability of the information from such an overview may prove invaluable when making these treatment decisions. Overviews also raise important clinical issues by highlighting different opinions regarding treatment. By initiating international collaborative ventures they provide a forum for debate which can lead to a useful exchange of ideas between trialists which may then be formulated as protocols for future studies.

The value of this approach has been demonstrated recently by the Early Breast Cancer Trialists' Collaborative Group which has led to the increased use of adjuvant therapy in patients with this disease. As a result it is estimated that an additional 1,000 lives per year may be saved in the UK alone (Early Breast Cancer Trialists' Collaborative Group, 1988; Editorial, 1989). Similarly, there are about 3,000 cases of advanced ovarian cancer diagnosed annually so that a 5% (from 20 to 25%) improvement in the 2-year survival rate could *potentially* 'save' 150 of these lives while a 10% improvement would save 300. Worldwide these figures mean that many thousands of deaths would be prevented each year. This would have a considerable impact on public health.

The results of this overview will give clinicians a more complete knowledge of the effects of the treatments they prescribe. This must be to the ultimate benefit of both clinician and patient.

Members of MRC Gynaecological Cancer Working Party: G. Blackledge, C.H. Buckley, S. Dische, D. Guthrie, P. Harper, R. Hunter, C.A.F. Joslin, A.H. Laing, R. Leonard, K.R. Peel, P.F. Schofield, F. Sharp, J.H. Shepherd, R.P. Symonds, V.R. Tindall, J. Tobias & C.J. Williams (Chairman).

### References

BEGG, C.B. & BERLIN, J.A. (1989). Publication bias and dissemination of clinical research. *J. Natl Cancer Inst.*, **18**, 107.

BERAL, V. (1987). Epidemiology. In *Ovarian cancer: the way ahead. Proceedings of the Seventeenth Study Group of the Royal College of Obstetricians and Gynaecologists in Conjunction with the Helene Harris Memorial Trust.* The Royal College of Obstetricians and Gynaecologists: London.

BRODOVSKY, H.S. (1984). Comparison of melphalan with cyclophosphamide, methotrexate and 5-flourouracil in patients with ovarian cancer. *Cancer*, **53**, 844.

CHALMERS, T.C., BLOCK, J.B. & LEE, S. (1972). Controlled studies in clinical cancer research. *N. Engl. J. Med.*, **287**, 1091.

CODY, M.M. & SLEVIN, M.L. (1989). Treatment decisions in advanced ovarian cancer. *Br. J. Cancer*, **60**, 155.

EARLY BREAST CANCER TRIALISTS' COLLABORATIVE GROUP (1988). Effects of adjuvant tamoxifen and cytotoxic therapy on mortality in early breast cancer. *N. Engl. J. Med.*, **329**, 1681.

EDITORIAL (1989). Adjuvant systemic treatment for breast cancer meta-analysed. *Lancet*, **i**, 80.

GRIFFITH, C.T. (1975). Surgical resection of bulk tumour in the primary treatment of ovarian carcinoma. *Natl Cancer Inst. Monogr.*, **42**, 101.

SIMES, R.J. (1986). Publication bias: the case for an international registry of clinical trials. *Clin. Oncol.*, **4**, 1529.

YOUNG, R.C., CHABNER, B.A. & HUBBARD, S.P. (1978). Prospective trial of melphan (L-PAM) versus combination chemotherapy (Hexa-CAF) in ovarian adenocarcinoma. *N. Engl. J. Med.*, **299**, 1261.